# Management of Acute Cancer Pain in Asia: An Expert Opinion on the Role of Tramadol/Dexketoprofen Fixed-Dose Combination

**DOI:** 10.7759/cureus.35770

**Published:** 2023-03-04

**Authors:** Jeffrey Tuan, Edward H Wang, Jose Rhoel C De Leon, Marvin Jonne Mendoza, Giustino Varrassi

**Affiliations:** 1 Division of Radiation Oncology, National Cancer Centre Singapore, Singapore, SGP; 2 Department of Orthopedics, College of Medicine - Philippine General Hospital, University of the Philippines Manila, Manila, PHL; 3 Department of Surgery, Capitol University Medical Center, Cagayan de Oro, PHL; 4 Cancer Institute, St. Luke's Medical Center - Global City, Manila, PHL; 5 Research, Paolo Procacci Foundation, Roma, ITA

**Keywords:** asia, asian patients, opioids, dexketoprofen, tramadol, fixed-dose combinations, multimodal analgesia, cancer pain, pain

## Abstract

Most patients experience acute cancer pain at some stage throughout their cancer journey. When inadequately managed, cancer pain has devastating consequences for the patient’s quality of life.

The suboptimal management of cancer pain in Asia is mainly driven by over-regulation and limited access to opioids. Concerns about adverse events and addiction have resulted in a negative perception of this group of drugs among physicians, as well as patients. There is a need to optimize the management of cancer pain across the region, through the provision of an alternative treatment option that is simple to prescribe, convenient to administer and well tolerated by patients, which will increase patients' compliance and good results.

As recommended in many international guidelines, starting by the WHO analgesic ladder, cancer pain can be effectively managed with multimodal analgesia. Fixed-dose combinations (FDCs), in which two or more analgesic agents act synergistically to deliver a broad spectrum of pain relief, represent an effective and convenient option for delivering multimodal analgesia to patients with cancer pain. This is extremely well accepted by patients for several reasons.

Any multimodal pharmacological approach to pain management should be based on the potentiality to block pain at different levels and to reduce the dosages of single analgesics, reducing their side effects. Hence, the use of NSAIDs, combined with other analgesics, is the general basis of multimodal pain management. If NSAIDs are combined with tramadol, a weak opioid that has per se a multimodal analgesic efficacy, it may be ideal. The tramadol/dexketoprofen FDC combines the centrally acting weak opioid with a peripherally acting NSAID to deliver rapid-onset, long-lasting analgesia, which has been proven efficacious and safe in the management of moderate-to-severe acute pain in the postoperative setting.

This expert opinion explores the role of tramadol/dexketoprofen FDC in the management of patients with moderate-to-severe acute cancer pain. It is essentially based on the incredibly high amount of existing data on the use of the drug, and on the long-lasting experience of the experts in pain management of cancer patients participating in the advisory panel.

## Introduction and background

Cancer pain is a common yet burdensome part of life for cancer patients, particularly in the advanced stages of the disease [1-3]. More than one-third of cancer patients have pain at the time of diagnosis [4]. This prevalence increases with advancing stages, to as much as 90% in patients with bone metastases [5]. At some stage in their cancer journey, most patients will experience acute pain. As many as 40-50% of patients with cancer pain experience moderate-to-severe acute pain [4].

When inadequately managed, cancer pain can have devastating consequences for a patient’s quality of life, causing emotional distress and anxiety, functional limitations, peripheral neuropathies, sleep disturbance and social withdrawal [1,6-9]. It can also cause treatment delay or withdrawal.

Recent studies suggest that there is suboptimal cancer pain management across Asia [1,10-14]. Expert insight gathered from clinicians in Southeast Asia suggests that cancer pain is generally undermanaged [1,12,14]. Access to strong opioids is significantly impaired in several Southeast Asian countries due to over-regulation and limited opioid formularies. This, together with concern about the adverse effects of strong opioids, has driven a negative perception of opioid treatments among clinicians, as well as patients [1,13,14].

Clinicians are hesitant to prescribe strong opioids due to the perceived complexities associated with their administration, and patients are reluctant to take strong opioids due to fear of addiction and other side effects or because they associate them with end-of-life care [1,11,12,14]. Similar findings were observed in China, with restrictive regulation on strong opioids and concerns about addiction, tolerance and side effects impacting the access and use of strong opioids [10,14]. As a result, many Asian patients with moderate or severe cancer pain do not receive adequate treatment to address their needs [1,10-14].

These findings suggest that there is an urgent necessity to optimize the management of cancer pain across the region. This should be obtained through the provision of an alternative treatment option that is simple to prescribe, convenient to administer and well tolerated by patients.

## Review

Multimodal analgesia for effective cancer pain management

Effective management of cancer pain requires an adequate understanding of its pathogenesis and the interventions available to treat it [15]. In this article, the term "cancer pain" is used to refer to both the pain caused by the cancer itself and the pain associated with treatments and/or interventions. Cancer pain may be caused by both the cancer itself and the associated treatments and/or interventions. It arises due to tissue damage, invasion of a tumor into somatic tissue with inflammation or ischemia and nerve compression or infiltration [16,17]. In general, it is a mixed mechanism pain - rarely presenting itself as a pure somatic, visceral or neuropathic pain [18,19]. The complex overlaps of different types of pain act concurrently to cause pain in the same area of the body [18,19].

There are two main mechanistic categories underlying cancer pain: nociceptive and neuropathic pain [16]. Nociceptive cancer pain, which can be classified further as somatic or visceral, results from the stimulation of nociceptors on normal sensory nerve endings [20]. This stimulation of nociceptors is caused by real or threatened damage to non-neural tissue [21].

Somatic nociceptive pain can be superficial or deep, depending on its site of origin, and is generally well localized to the site of pain stimuli [16]. Causes of somatic nociceptive cancer pain include bone metastases and malignant ulcers.

Visceral nociceptive pain involves pain stimuli in internal organs, such as the bowel, liver and lungs. It is poorly localized, with referred pain and autonomic effects including sweating, nausea and changes in blood pressure. Causes of visceral nociceptive cancer pain include ureteric colic and hepatic capsule stretch [16,20]. Neuropathic cancer pain results from injury or compression of the nerves or other structures of the nervous system. It is caused by direct damage to the nerves from a tumor or through post-treatment neuralgia following surgery, radiotherapy and chemotherapy [9,16,21,22]. Regardless of its underlying mechanisms, cancer pain can be broadly categorized as acute or chronic. Most acute cancer pain can be directly attributed to a diagnostic test or treatment, such as a lumbar puncture or surgical debulking procedures. However, there are some disease-related causes of acute cancer pain, such as pathologic bone fracture, tumor hemorrhage and acute visceral pain due to an obstruction or perforation [23].

Chronic cancer pain is most often due to the tumor itself but can also result from post-treatment neuralgia [22,23].

Consideration of the underlying mechanisms of a patient’s cancer pain, as well as its nature as chronic or acute, can assist in the selection of appropriate analgesic treatment [16].

**Figure 1:**
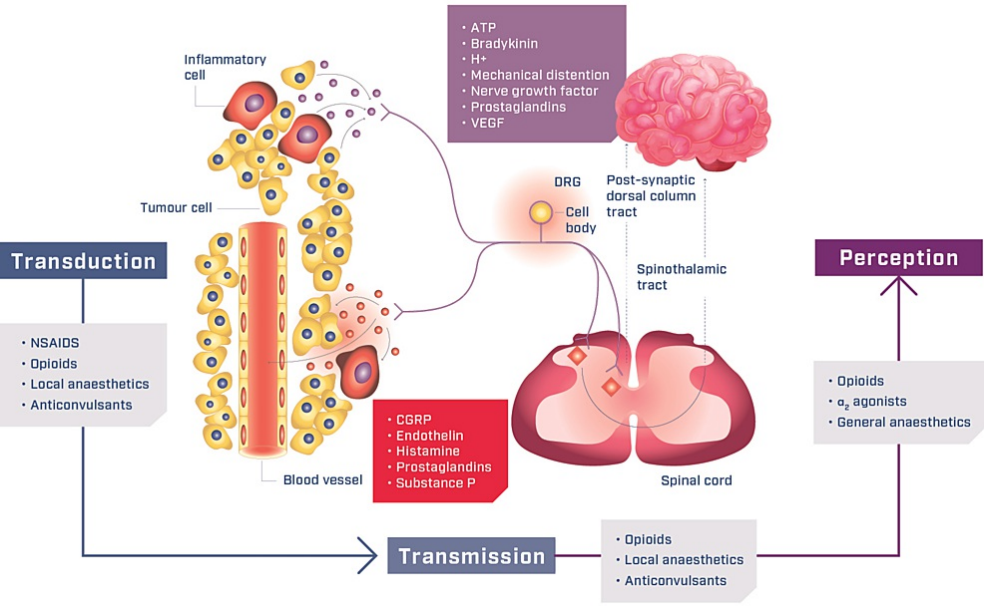
Therapeutic targets in multimodal analgesia in the cancer pain pathway Adapted from Mantyh PW et al.: Molecular mechanisms of cancer pain. Nature Reviews Cancer 2002; 2:201-209 and Berry PH et al.: Pain: Current understanding of assessment, management and treatments. National Pharmaceutical Council and the Joint Commission for the Accreditation of Healthcare Organizations 2001 [24]. ATP, adenosine triphosphate; CGRP, calcitonin gene-related peptide; DRG, dorsal root ganglion; H+, hydrogen ions; NSAIDs, non-steroidal anti-inflammatory drugs

Cancer pain can be effectively managed with multimodal analgesia-the use of more than one analgesic medication with different mechanisms of action to obtain additive or synergistic effects [2,15,16]. Combining analgesics with differing mechanisms of action and pharmacokinetic profiles can target pain at different points on the pain pathway (Figure 1) [24]. Thus, providing a broader spectrum of relief for both nociceptive and neuropathic pain, while minimizing the side effects associated with high doses of a single analgesic treatment [25]. A typical multimodal treatment regimen for the management of cancer pain may include varying combinations of NSAIDs or acetaminophen with an opioid analgesic appropriate to the severity of the pain.

Multimodal analgesia forms the backbone of cancer pain management across multiple international clinical practice guidelines [2,15,20,26-28].

The most widely accepted algorithm for the multimodal treatment of cancer pain is the WHO analgesic ladder [20]. First published in 1986 by a WHO expert committee, the ladder was based on the premise that healthcare professionals should learn to "use a few drugs well" [20]. Since its initial publication, it has been widely disseminated and accepted around the world [20]. While clinical practice continues to evolve, it remains a useful educational tool and guide for practice.

The WHO analgesic ladder recommends that adults and adolescents with mild pain related to cancer be initiated on an NSAID or acetaminophen, associated or not with adjuvant drugs. If pain is not well controlled, or for those with moderate and severe cancer pain, therapy should be escalated to include a "weak opioid" and subsequently a "strong opioid," if required [20].

The concept of a stepped approach to multimodal analgesia dependent on pain severity for cancer pain is supported across international pain guidelines (Table 1) [2,15,20,26-28].

**Table 1:** International clinical practice guideline recommendations on the management of cancer pain ^a^ World Health Organization Guidelines for the pharmacological and radiotherapeutic management of cancer pain in adults and adolescents [20] ^b^ European Society of Medical Oncology Clinical Practice Guidelines for the management of cancer pain in adults [2] ^c^ National Comprehensive Cancer Network Clinical Practice Guidelines in Oncology. Adult Cancer Pain, Version 2.2022 [15] ^d^ British Pain Society cancer pain management perspective [26] ^e^ Malaysian Association for the Study of Pain Clinical Practice Guidelines for the management of cancer pain [27]

Clinical practice guideline recommendations in cancer pain	WHO^a^	ESMO^b^	NCCN^c^	BPS^d^	MASP^e^
An oral route of administration is preferred, when appropriate for the patient	√	√	√	√	√
NSAIDs or acetaminophen should be used for initial cancer pain management	√	√	√	√	√
The addition of a weak opioid is accepted for cancer pain not adequately controlled with NSAID or acetaminophen alone	√	√	√	√	√

The *European Society of Medical Oncology* (ESMO) *Clinical Practice Guidelines on the Management of Cancer Pain* supports the use of NSAIDs, alone or in combination with opioids, for mild-to-moderate pain. It notes a role for tramadol in this multimodal approach [2].

Similarly, the *National Comprehensive Cancer Network* (NCCN) *Guidelines for Adult Cancer Pain* supports the use of NSAIDs or other non-opioid analgesics before consideration of opioids. The need for escalation to multimodal analgesia including strong opioids should be considered on an as-needed basis [15].

The *Malaysian Association for the Study of Pain* (MASP) also follows a stepped approach to pain management. NSAIDs and other non-opioid analgesics are recommended for mild pain, with the introduction of a weak opioid, such as tramadol or codeine recommended for moderate pain [27].

Recently, the *American Society of Clinical Oncology* (ASCO) published guidelines for the use of opioids in cancer pain that recommended the continuation of analgesics, such as NSAIDs, if these agents provide additional analgesia once opioids have been initiated [28].

The role of fixed-dose combinations to optimize multimodal analgesia in cancer pain

Fixed-dose combinations (FDCs) can be an effective and convenient method for delivering multimodal analgesia to patients with cancer pain. Typically, FDCs combine two or more analgesic agents that work synergistically to deliver a broad spectrum of pain relief at lower and better-tolerated doses than those of the single agents used alone. This pharmacological synergism is achieved through the combination of agents with differing mechanisms of action, for example, peripheral and central analgesia. When combined at lower doses, their mechanisms create an increased analgesic effect with a lower efficacy-to-safety ratio [25,29]. The potentiality to achieve this increased analgesic effect through the combination of a non-opioid analgesic and a weak opioid analgesic is of particular benefit in the Asia Pacific region, where strict regulatory requirements limit access to strong opioid treatment options. FDCs provide a strategy to simplify prescription requirements for clinicians in these countries.

These easy-to-administer oral formulations have further benefits to patients, including reduced pill burden and the potential for enhanced patient confidence and adherence [30,31]. The convenience benefits can be especially relevant for cancer patients for whom polypharmacy and the potential for poor treatment compliance is a considerable concern [32].

Examples of FDC analgesics available for the management of cancer pain include codeine-acetaminophen, tramadol-acetaminophen, oxycodone-acetaminophen and tramadol-dexketoprofen [33]. Each combines a non-opioid analgesic with an opioid in an opioid-sparing formulation.

International cancer pain guidelines consider both acetaminophen and NSAIDs as appropriate non-opioid analgesic choices for cancer pain of any severity [2,20]. However, the combination of an opioid with an NSAID may be considered optimal due to the anti-inflammatory properties of the latter.

NSAIDs are a heterogeneous group of drugs, encompassing nonselective NSAIDs (nsNSAIDs) and COX-2 selective inhibitors (coxibs) [25]. NSAIDs act to reduce prostaglandin synthesis through the inhibition of COX enzymes, resulting in the reduction of inflammation and pain [34]. A 2017 Cochrane review of 11 studies of oral NSAIDs in adults with cancer pain demonstrated that moderate or severe cancer pain was reduced to mild pain in up to 51% of patients receiving NSAID treatment [2,35].

Dexketoprofen is one example of an NSAID that is available in an FDC for the management of cancer pain. As a traditional nsNSAID, dexketoprofen works through the inhibition of both COX-1 and COX-2. It has peripheral and central mechanisms, reducing the synthesis of prostaglandins and subsequent pain and inflammation at the site of cancer pain, while also acting to reduce pain response in the central nervous system [30]. The S(+)-enantiomer of ketoprofen, dexketoprofen, has equal analgesic activity with a faster onset at half the dose of the racemic ketoprofen [25,30]. This fast onset of therapeutic effect results from a high solubility that enhances its bioavailability. Fast absorption also lowers the potential for gastric ulceration, resulting in an improved tolerability profile compared with other NSAIDs.

Dexketoprofen 25 mg was demonstrated to provide effective and well-tolerated relief for bone cancer pain in a randomized, double-blind parallel group study of 115 patients with a pain index rating ≥10. Following one week of treatment, patients receiving dexketoprofen reported a significantly lower pain index rating than those receiving the comparator drug, ketorolac 10 mg (8.5±2.3 vs. 9.7±2.9, P=0.04). More than half of the dexketoprofen patients achieved a pain intensity <30 mm on the 100 mm visual analog scale. Treatment-related adverse events and treatment withdrawal due to adverse events were lower in the dexketoprofen group than in the ketorolac group. One case of gastrointestinal hemorrhage was considered related to ketorolac [36].

Tramadol - a centrally acting μ-opioid receptor agonist and serotonin/norepinephrine reuptake inhibitor (SNRI) has been used effectively to treat moderate-to-severe pain since the 1970s and is the most commonly used weak opioid for the management of cancer pain all over the world, including Asia [11,37]. It is metabolized by the liver enzyme CYP2D6. In patients with a CYP2D6 deficiency, it can be challenging to obtain an adequate analgesic effect. Conversely, patients who are ultra-rapid metabolizers risk developing side effects of opioid toxicity even at commonly prescribed doses. Research indicates low percentages of poor metabolizers (1-2%) and ultra-rapid metabolizers (1.2-2%) within Asian populations [38,39]

Endorsed as an appropriate weak opioid option in multiple cancer pain clinical practice guidelines, tramadol has demonstrated adequate relief for cancer pain in clinical trials [2,15,27]. In a 2008 double-blind, comparative trial versus a hydrocodone/acetaminophen combination, one dose of tramadol relieved pain in >60% of patients, which was comparable and non-inferior to the combination treatment [40]. Tramadol has also been assessed as providing adequate relief to moderate cancer pain in clinical trials versus controlled-release morphine [41-43].

Tramadol’s long duration of action is an ideal complement to the rapid onset of pain relief achieved with dexketoprofen [30]. A tramadol 75 mg and dexketoprofen 25 mg fixed-dose combination (TRAM/DKP FDC) was introduced in Europe in 2016 and across the Asia Pacific region in 2018, where its use is limited to a maximum of five days. It is indicated for the relief of moderate-to-severe acute pain, such as that seen with cancer pain [30,31]. The TRAM/DKP FDC may represent an additional option in the analgesic armamentarium for the short-term management of acute cancer pain.

Appropriate patient selection and understanding of each drug component’s mechanisms of action could maximize the potential of the combination while being wary of the possible adverse reactions.

The efficacy and safety of the TRAM/DKP FDC for moderate-to-severe pain have been well demonstrated in the postoperative acute pain setting. The TRAM/DKP FDC demonstrated superior pain relief compared with placebo, and tramadol and dexketoprofen monotherapy in abdominal hysterectomy, total hip replacement and third molar extraction [44-46]. At the moment, it is also studied for acute low back pain [47].

The superiority of the TRAM/DKP FDC has also been assessed in one head-to-head randomized, double-blind single-dose trial versus a tramadol 75 mg plus paracetamol 650 mg (TRAM/PARA) combination or placebo in postoperative dental pain resulting from third molar extraction [48]. The TRAM/DKP FDC demonstrated superior total pain relief over 6 hours (TOTPAR6) compared with TRAM/PARA, with significantly more patients treated with the TRAM/DKP FDC noting pain relief within 30 minutes of taking a dose [48].

The safety and tolerability of the TRAM/DKP FDC in these trials are in line with that observed in previous clinical experience with tramadol and dexketoprofen monotherapy. Vomiting, nausea and dizziness are the most frequently reported adverse reactions, with most being mild-to-moderate in intensity [44-46,48].

The TRAM/DKP FDC has also demonstrated tolerability in Asian patients. A 2020 case series outlined the experience of 13 Asian patients treated with the TRAM/DKP FDC for pain management in the postoperative setting. All cases reported adequate pain relief with the TRAM/DKP FDC that was well tolerated, with no discontinuations during the treatment period [49].

Experts’ opinion on the role of the tramadol/dexketoprofen fixed-dose combination in cancer pain management

**Figure 2:**
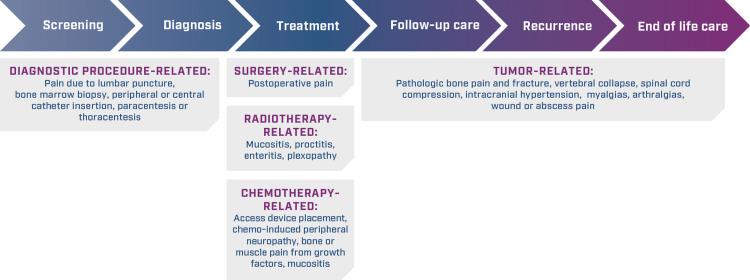
Origination of acute pain throughout the cancer patient journey

Acute cancer pain can occur at any stage of the cancer pathway, and thus, there is a potential role for the TRAM/DKP FDC for treating moderate-to-severe pain in various clinical scenarios (Figure 2) [23].

Regardless of the origin of acute cancer pain, management should always be individualized to the needs of the patient. Adequate assessment and reassessment of pain are recommended to ensure the appropriate continuation or discontinuation of pain medication.

The choice of an analgesic regimen to manage cancer pain is driven primarily by a patient’s rating of the severity of their pain. It is common practice to commence patients on acetaminophen or NSAIDs for mild pain and to follow the WHO analgesic ladder by introducing a multimodal regimen of weak or strong opioids with increasing pain severity.

**Figure 3:**
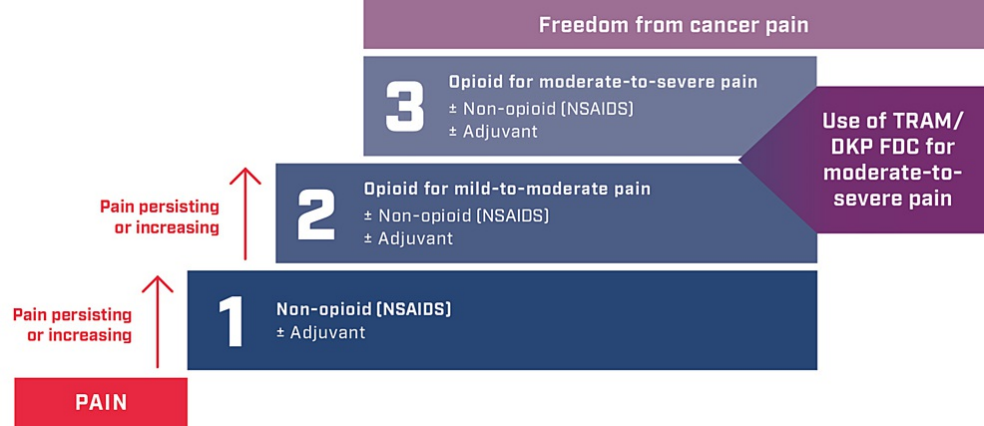
Potential use of TRAM/DKP FDC in the context of the WHO analgesic ladder TRAM/DKP FDC, tramadol 75 mg and dexketoprofen 25 mg fixed-dose combination

The TRAM/DKP FDC may fit the recommendations of the second and third steps of the WHO analgesic ladder (Figure 3) and provides a potential option for the short-term (up to five days) relief of acute moderate-to-severe cancer pain before progressing to strong opioids.

The synergistic relationship of tramadol and dexketoprofen, which is pharmacologically well demonstrated [50], provides proven relief to moderate-to-severe pain, at lower and better-tolerated doses than if the single analgesic agents were used alone [44-46,48].

The efficacy of the TRAM/DKP FDC is well demonstrated for acute pain in the postoperative setting, with evidence of its superiority over a tramadol/acetaminophen combination [48]. Its tolerability is also well accepted, with case studies supporting its use in Asian patients [49].

The simple, oral formulation overcomes barriers of perceived complexity with strong opioid therapies, reducing pill burden and enhancing compliance for patients navigating polypharmacy.

The wide use of the TRAM/DKP FDC in the postoperative setting, and its expanding use in other areas of moderate-to-severe acute pain including low back pain, makes the combination readily accessible and available on formularies across the region.

Considering the opioid hesitancy prevalent among Asian clinicians and patients, driven by a fear of the adverse effects and stigma of strong opioid use, the TRAM/DKP FDC represents a more appealing and accessible treatment option for moderate-to-severe cancer pain, especially in this area of the world, where the genetic setting is providing a reduced incidence of side effects after the administration of tramadol [49].

The TRAM/DKP FDC may become a convenient multimodal analgesic option for the short-term (five days) management of patients with acute cancer pain who do not desire to escalate to treatment with strong opioids and for those currently taking strong opioids who are seeking a change in treatment to reduce their risk of adverse effects.

Multidisciplinary collaboration and/or subsequent referral to a pain specialist may be warranted in patients whose acute cancer pain episode is not adequately controlled.

## Conclusions

Most patients with cancer will experience acute pain at some stage during their cancer experience. Inadequately managed moderate-to-severe acute cancer pain can have a significant impact on a patient’s life, causing distress, anxiety, functional limitations, sleep disturbance and social withdrawal. Multimodal analgesia is an effective way to target the mixed mechanisms underlying moderate-to-severe acute cancer pain. FDCs offer a convenient and accessible approach to multimodal analgesia that reduces a patient’s pill burden, simplifies prescription requirements and, in turn, overcomes some of the limitations and hesitancy to accessing opioids seen across the Asian region.

The TRAM/DKP FDC is well proven to provide effective relief from moderate-to-severe acute pain, for which it is indicated for up to five days of use. The pharmacological synergism between the centrally acting tramadol and the peripherally acting dexketoprofen delivers more effective analgesic activity at lower and better-tolerated doses than either agent used alone. The rapid onset of dexketoprofen means that patients with acute cancer pain can achieve effective relief in a timely manner, and the long-acting duration of tramadol means this pain will remain adequately managed for longer. For carefully selected and suitable patients, the TRAM/DKP FDC might be an additional short-term treatment option to the multimodal analgesia treatment armamentarium for moderate-to-severe acute cancer pain for up to five days of use.
